# Sensitive detection of miR-21 and miR-25 in gastric adenocarcinoma patient serum using a SERS sensor based on AuNT and enzyme cleavage strategy†

**DOI:** 10.1039/d4ra08761e

**Published:** 2025-02-10

**Authors:** Gaoyang Chen, Ming Tan, Long Jia, Yayun Qian, Hongjun Yin, Jinhua Zhu

**Affiliations:** a Department of Gastroenterology, Yangzhong People's Hospital Zhenjiang 212200 P. R. China 541292188@qq.com; b Department of Oncology, The Affiliated Taizhou Second People's Hospital of Yangzhou University Taizhou 225300 China; c Institute of Tumour Prevention and Control, Yangzhong People's Hospital Zhenjiang 212200 P. R. China yzrykjk@163.com; d Department of General Surgery, Yangzhong People's Hospital Zhenjiang 212200 P. R. China; e Institute of Translational Medicine, Medical College, Yangzhou University Yangzhou 225001 P. R. China

## Abstract

MicroRNA (miRNA) detection has significant application value for early cancer diagnosis. In this study, a surface-enhanced Raman scattering (SERS) sensor was developed for detecting miR-21 and miR-25 in the serum of Gastric adenocarcinoma (GAC) patients. The sensor was constructed using arrays of Au trioctahedral nanoparticles (AuNT) and enzyme cleavage techniques. The AuNT was obtained by self-assembly at the oil–water interface, and the Cy5-labeled miR-21 and 5-FAM-labeled miR-25 complementary single-stranded ssDNA-21 and ssDNA-25 were connected with the AuNT to form the SERS sensor. When miR-21 and miR-25 were present, ssDNA-21 and ssDNA-25 were paired and hybridized to form miR-21-ssDNA-21 and miR-25-ssDNA-25 double strands. Duplex-specific nuclease (DSN) could act on the DNA phosphodiester bond in the double strand, causing Cy5 and 5-FAM to be far away from the AuNT, which resulted in a reduction of the SERS signal. In the range of 10 aM to 1 pM, the logarithm of miR-25 concentration was linearly related to the intensity of the characteristic peak of 5-FAM at 1178 cm^−1^, and the limit of detection (LOD) was determined to be 8.12 aM. The logarithm of miR-21 concentration was linearly related to the characteristic peak intensity of Cy5 at 1367 cm^−1^, and the LOD was determined to be 4.29 aM. Furthermore, the accuracy of the SERS sensor for the detection of miR-21 and miR-25 in clinical serum samples was evaluated using real-time quantitative polynucleotide chain reaction (qRT-PCR) technology as the gold standard. The relative errors of the two methods miR-21 in healthy people and gastric adenocarcinoma patients were 1.71% and −2.40%. The relative errors of miR-25 were 2.74% and −2.67%. There was no significant difference between the two methods, and the expression levels of miR-21 and miR-25 in the serum of GAC patients were found to be higher than those in healthy individuals. Consequently, this method offers a reliable solution for the early diagnosis of gastric cancer.

## Abstract 2

MicroRNA (miRNA) detection has significant application value for early cancer diagnosis.

## Graphical abstract

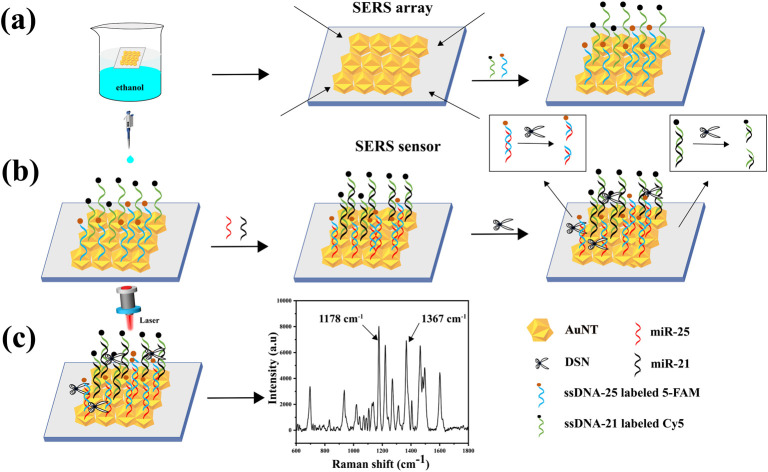

## 1. Introduction

Gastric adenocarcinoma (GAC) has a high incidence rate and mortality. It is a common gastrointestinal malignant tumor in clinical practice and one of the major causes of cancer death worldwide.^1^ Early gastric adenocarcinoma (EGAC) is limited to the mucosa and submucosa, lacking typical clinical symptoms.^2^ This results in a lower diagnostic rate and five-year survival rate. Therefore, early diagnosis of GAC patients can help improve their five-year survival rate. Accordingly, the early detection of GAC is pivotal for enhancing the five-year survival rate of affected individuals. Currently, the clinical diagnosis of GAC predominantly hinges on endoscopic evaluation and histopathological analysis.^3^ Nonetheless, these diagnostic modalities are associated with significant costs, invasiveness, and necessitate a high level of technical proficiency from medical practitioners. Consequently, there is an urgent need to develop a non-invasive, cost-effective, and expeditious diagnostic approach for the early detection of gastric cancer, facilitating prompt identification and optimal therapeutic intervention for patients afflicted with this malignancy.

The short non-coding RNA molecule microRNA (miRNA), which are small non-coding RNA molecules ranging from 20 to 40 nucleotides in length, play a crucial role in post-transcriptional regulation by binding to complementary sequences within their target messenger RNAs. This interaction can lead to mRNA degradation or translational repression, thereby modulating gene expression.^4,5^ MiRNAs have emerged as pivotal modulators in a plethora of cellular processes, including cell differentiation, proliferation, and apoptosis.^6,7^ Moreover, their dysregulation has been implicated in the pathogenesis of a myriad of diseases, with a significant focus on oncology, where they can function as either oncogenes or tumor suppressors, depending on the context. In the realm of oncology research, miRNAs have emerged as pivotal regulators in the pathogenesis of gastric cancer. Notably, miR-21 and miR-25 have been identified as significantly upregulated in GAC tissues compared to their levels in healthy controls, suggesting a potential role in tumorigenesis.^8,9^ MiR-21 has been implicated in the promotion of gastric cancer cell growth and invasiveness through the suppression of tumor suppressor genes, such as phosphatase and tensin homolog (PTEN) and programmed cell death protein 4 (PDCD4).^10^ These proteins are known to exert inhibitory effects on tumor progression, and their downregulation by miR-21 may contribute to the malignant phenotype of gastric cancer cells. Meanwhile, miR-25 has been demonstrated to exert its regulatory effects by directly targeting the early growth response protein 2 (EGR2), which is an essential transcription factor involved in the control of gene expression.^11^ Currently, the methodologies employed for the detection of miR-21 and miR-25 encompass northern blotting, real-time quantitative reverse transcription polymerase chain reaction (qRT-PCR), and microarray analysis.^12–14^ However, these techniques are not without their limitations, which include diminished sensitivity, elevated costs, stringent technical proficiency requirements, and intricate experimental procedures.

Surface-Enhanced Raman Scattering (SERS) is a sophisticated analytical technique employed for the detection and identification of molecular species.^15,16^ The signal enhancement in SERS is predicated upon two principal mechanisms: the electromagnetic field enhancement effect and the chemical enhancement effect. At specific wavelengths, the interaction between the incident light and the free electrons on the metal surface engenders a localized amplification of the electromagnetic field. This phenomenon is denoted as Localized Surface Plasmon Resonance (LSPR).^17,18^ The LSPR effect is particularly pronounced in certain microenvironments of metal nanostructures, such as interstitial regions between nanoparticles or the apex of nanostructures, which are designated as “hotspots”. These hotspots are characterized by an intense electromagnetic field enhancement, which substantially augments the SERS signal.^19,20^ The synergistic action of these two effects confers upon SERS its exceptional sensitivity and the ability to provide molecular fingerprinting. Consequently, SERS has been widely adopted across various domains, including biomedical diagnostics, food safety assessments and environmental monitoring.^21–23^ Au trioctahedral nanoparticles (AuNT) represents a novel class of nanocomposite materials that have garnered significant interest in the scientific community owing to their distinctive optical characteristics and favorable biocompatibility profiles.^24^ AuNT possess a unique geometric shape, with their abundant sharp corners acting as “hotspot” regions that significantly enhance the LSPR effect. Moreover, there is also a more pronounced signal enhancement between adjacent AuNT. AuNT exhibit special optical and plasmonic properties due to their unique triangular anisotropy. The three sharp vertices of AuNT exhibit a significant hotspot enhancement effect, which is manifested in the optical properties as strong LSPR. The LSPR of AuNT can be extensively regulated by varying its size, edge length, thickness, and proportion. AuNT can be easily surface-modified with compounds such as proteins, peptides, oligonucleotides, *etc.*, while maintaining their optical properties. AuNT have been developed in chemical biology and sensing applications due to their unique physical properties and good biocompatibility.^25^ The detection methods of miRNAs based on SERS technology mainly include the “sandwich” sandwich strategy, the signal switch strategy and the signal amplification method of chain hybridization reaction.^26–28^ While these methods are capable of meeting the requirements for highly sensitive detection of trace substances, they are hindered by the drawbacks of complex procedures and cumbersome steps. The “sandwich” approach within the realm of SERS is a widely employed methodology in bioanalytical assays, specifically within the context of antibody-mediated sandwich immunoassays. This technique has been effectively utilized for the detection of a diverse array of biomarkers, encompassing oncological indicators, bacterial antigens, and viral pathogens.^29–31^ However, it is noteworthy that the assay procedure is associated with extended durations of analysis. The enzyme cleavage strategy makes clever use of duplex-specific nuclease (DSN), which can accurately recognize and cleave the DNA phosphodiester bonds in the nucleic acid double-stranded nucleic acid formed by the target miRNA and its complementary single-stranded DNA (ssDNA), thus achieving highly sensitive and specific detection of miRNAs, which is easier to operate.^32–34^

**Fig. 1:**
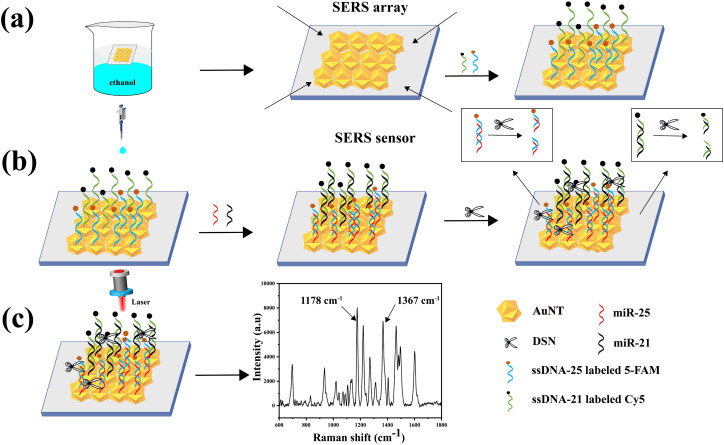
(a) AuNT self-assembled at the oil–water interface to form a tightly packed monolayer array. Clean hydrophilic silicon wafers were immersed in the solution, and then brought into parallel contact with the array to transfer the AuNT onto the wafers. Subsequently, the silicon wafers were immersed in a solution containing a mixture of TCEP and ssDNA-21 and ssDNA-25 to prepare the SERS sensor. (b) MiR-21 and miR-25 were added dropwise to ssDNA-21 and ssDNA-25. This was followed by the dropwise addition of DSN, which cleaved the DNA phosphodiester bond in the nucleic acid double-stranded nucleic acid. (c) SERS spectra of miR-21 and miR-25.

In this research, a SERS sensor based on AuNT and an enzymatic shearing approach was created to measure the expression levels of miR-21 and miR-25 in the serum of GAC patients. The detection mechanism is depicted in Fig. 1. The procedure started with synthesizing AuNT, which was then used to create the SERS sensor. The AuNT was self-assembled at the cyclohexane/water interface, commonly referred to as the oil–water interface. The monolayer array formed at this interface was then transferred onto a hydrophilic silicon wafer using a pipette, resulting in the formation of an AuNT array. Subsequently, the Raman reporter molecule Cy5-labeled miR-21 complementary single-stranded ssDNA-21 and 5-FAM-labeled miR-25 complementary single-stranded ssDNA-25 were conjugated to the surface of AuNT through Au–S bonds. This step culminated in the fabrication of SERS sensors capable of specifically interacting with miR-21 and miR-25. In the presence of miR-21 and miR-25, miR-21 was hybridized with ssDNA-21 in the SERS sensor to form a miR-21–ssDNA-21 double strand, and miR-25 was hybridized with ssDNA-25 in the SERS sensor to form a miR-25–ssDNA-25 double strand. Afterwards, DSN was used to specifically break the DNA phosphodiester bond in the double strands. This enzymatic cleavage resulted in the signaling molecules 5-FAM and Cy5 being displaced from the surface of the SERS sensor, thereby diminishing the Raman signal. In this methodology, the intensity of the characteristic peaks of 5-FAM and Cy5 at 1178 cm^−1^ and 1367 cm^−1^, respectively. It could be utilized to achieve highly sensitive detection of miR-21 and miR-25 in serum. The reduction in the Raman signals corresponded to the presence and concentration of the target miRNAs, providing a quantitative measure of miR-21 and miR-25 levels. This approach demonstrated the potential for precise and sensitive miRNA detection, which is crucial for the early diagnosis and monitoring of gastric cancer.

## 2. Research methods

### 2.1 Materials

Cetyl trimethylammonium chloride (CTAC), chloroauric acid (HAuCl_4_), 5-carboxyfluorescein (5-FAM), Cy5, ascorbic acid (AA), polyvinylpyrrolidone (PVP), 5,5-dithiobis-2-nitrobenzoic acid (DTNB), and phosphate-buffered saline (PBS) were obtained from Shanghai Bioengineering Co. The miR-21, miR-25, ssDNA-21, ssDNA-25, single-base mismatches (MT1-21 and MT1-25), tri-base mismatches (MT3-21 and MT3-25), and random nucleic acid sequences listed in Table 1 were also purchased from the same supplier. MilliQ Purifier-generated deionized water was utilized (resistivity 18 MΩ cm).

**Table 1:** Nucleic acid sequences used in the experiment

Name	Sequence (5′–3′)
miR-21	UAGACUUAUCAGACUGAUGUUGA
ssDNA-21	SH-TCAACATCAGTCTGATAAGACTA-Cy5
miR-25	CAUUGACACUUGUCUCGGUCUGA
ssDNA-25	SH-TCAGACCGAGACAAGTGACAATG-5-FAM
MT1-21	UAGACUUAUCACACUGAUGUUGA
MT1-25	CAUUGACACUAGUCUCGGUCUGA
MT3-21	TCAACATAAGTCAGAGAAGACTA
MT3-25	CAUCGACAGUUGACUCGGUCUGA
Random	GACACUAUCGAAUCCACUGGGU

### 2.2 Collection of samples

30 serum samples were obtained from healthy individuals (15 males and 15 females, average age 46 years) and 30 serum samples were collected from GAC patients (16 males and 14 females, average age 50 years). All serum specimens had been stored at −80 °C for testing. The serum samples in the experiment had been provided by the Yangzhong People's Hospital, and the subjects had all signed the informed consent form.

### 2.3 Synthesis of AuNT

0.195 g of CTAC was dissolved in 50 mL of deionized water, and then 50 mL of 0.6 mM HAuCl_4_ was slowly added to the solution under room temperature with gentle stirring. Following this, 2.45 mL of freshly prepared 0.15 M AA was promptly introduced to the mixture. The mixture was stirred gently and allowed to react at 30 °C for 20 min, during which the color of the mixture gradually changed from colorless to light pink. The solution was subsequently centrifuged at 7000 rpm for 10 min, followed by three washes with water to eliminate CTAC from the precipitate. Finally, ultrapure water was added to fix the volume to 12 mL.

### 2.4 Preparation of SERS sensors

The clean silicon wafers were immersed in piranha solution (the concentration ratio of H_2_SO_4_ and 30% H_2_O_2_ was 7 : 3), heated in a water bath for 1.5 h, and then washed and dried with ultrapure water to obtain hydrophilic silicon wafers.^35^ After that, 2 mL of AuNT and 4 mL of *n*-hexane were sequentially added to the same dry beaker, followed by the rapid addition of 2 mL of ethanol to the system. After standing for three minutes, gold nanoparticles self-assembled at the oil–water interface to form a tightly packed monolayer array. Next, clean silicon wafers were immersed in the solution, and the wafers were brought into parallel contact with the arrays to transfer them onto the wafers. They were then dried in a desiccator for 10 min. Concurrently, twenty microliters of 1 M tris(2-carboxyethyl)phosphine (TCEP) solution was mixed with two hundred microliters of ssDNA-21 and ssDNA-25 (5 mM). TCEP reduced the disulfide bonds of sulfhydrylated DNA and improved the efficiency of the coupling reaction. After incubation at 37 °C for 12 h, ssDNA-21 and ssDNA-25 attached to the AuNT surface through Au–S bonds. After washing with PBS solution, the SERS sensor was successfully fabricated.

### 2.5 SERS measurement

The solution to be tested was added dropwise to the SERS sensor and incubated in a 37 °C incubator for 2 h to facilitate the formation of stable miR-21–ssDNA-21 and miR-25–ssDNA-25 double strands. Subsequently, DSN solution (15 U mL^−1^) was introduced dropwise onto the SERS sensor and allowed to incubate for 30 min. Following this, the sensor underwent several rinses with PBS buffer prior to SERS detection. The Raman spectrometer employed in the experiment featured an excitation wavelength of 785 nm, a laser intensity of 5 mW, and an exposure time of 10 s.

### 2.6 Instrumentation

Scanning electron microscope (SEM) images were obtained by running the S-4800II field emission scanning electron microscope (Hitachi, Japan) at 10 kV. Transmission electron microscopy (TEM) images were acquired by a transmission electron microscope (Philips Tecnai 12) running at 120 kV. High-resolution TEM (HRTEM) was obtained by emission transmission electron microscopy (JEOL JEM-2100Plus, Japan) at an accelerating voltage of 200 kV. The UV-vis spectrum was recorded by a Cary 5000 UV-vis spectrophotometer and processed by Oringin software. Raman spectra of all samples were collected by DXR xi micro-Raman spectrometer (Thermo Fisher, United States).

## 3. Results and discussion

### 3.1 Characterization of AuNT array

**Fig. 2:**
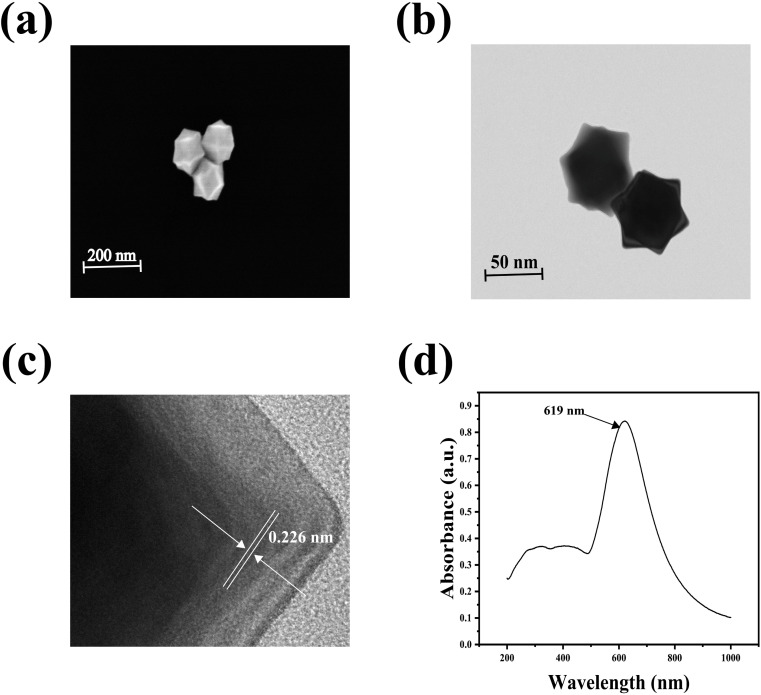
(a) SEM and (b) TEM images of AuNT, (c) HRTEM image of AuNT, (d) UV-vis absorption spectrum of AuNT.

Fig. 2(a) and (b) presented the SEM and TEM images of AuNT, illustrating the successful preparation of morphologically regular AuNT that were distributed in various angular orientations within the field of view. Fig. 2(c) was the HRTEM image of AuNT, depicting the lattice fringe spacing of gold nanoparticles at 0.226 nm. Fig. 2(d) showed that AuNT exhibited strong surface plasmon resonance absorption in the range of 500 to 800 nm in the UV-vis-NIR spectra, with a peak at 619 nm. The improvement was credited to the distinct shape of the synthesized AuNT, featuring many sharp edges serving as “hot spots” that significantly boosted the LSPR effect. Furthermore, a more noticeable signal enhancement was also observed among adjacent AuNT. Consequently, AuNT were selected as efficient SERS substrate.

**Fig. 3:**
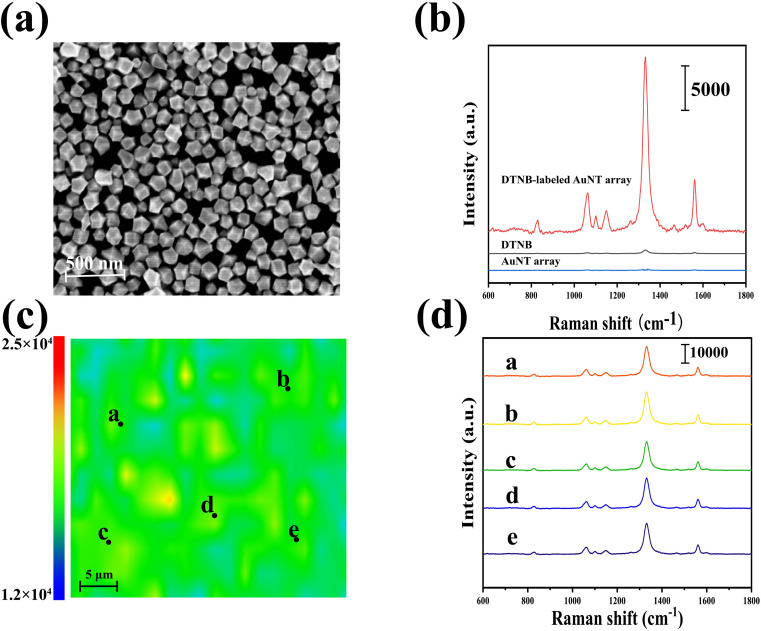
(a) SEM image of an AuNT array, (b) SEM image of an AuNT array, (b) SERS spectra of DTNB (10^−2^ M), DTNB (10^−8^ M)-labeled AuNT array and AuNT array, (c) SERS mapping of the DTNB (10^−8^ M)-AuNT array, (d) SERS spectra when 5 different points (a–e) were arbitrarily selected on the DTNB (10^−8^ M)-AuNT array.

Fig. 3(a) displayed the SEM image of self-assembled AuNT array. Fig. 3(b) compared the Raman signal characteristics of pure DTNB (10^−2^ M) with those of DTNB (10^−8^ M)-labeled AuNT array to assess the ability of AuNT array to enhance the signal. It was found that the unmodified pure DTNB displayed very low Raman signal intensity, whereas the characteristic Raman peaks of DTNB was significantly enhanced in the DTNB (10^−8^ M)-AuNT array. The concentration of DTNB used to plot the spectrum was 10^−8^ M, and the plotted area of the selected T-line was 50 × 50 μm^2^. Fig. 3(c) showed the SERS mapping of a DTNB (10^−8^ M)-AuNT array. When we randomly picked 5 different points from the DTNB (10^−8^ M)-AuNT array, as shown in Fig. 3(d), the measured SERS spectra were approximately similar, indicating that the AuNT array were in good agreement.

The enhancement factor (EF) for the AuNT array was determined using the formula EF = (*I*_SERS_/*C*_SERS_)/(*I*_RS_/*C*_RS_), where *I* and *C* denoted the Raman signal intensity and concentration of the analyte, respectively. The EF was determined based on the characteristic peak of DTNB at 1341 cm^−1^. When the concentration of *C*_SERS_ was 1 × 10^−8^ M and *C*_RS_ was 1 × 10^−2^ M, the calculated EF value of the assembled monolayer of AuNT was found to be 3.2 × 10^8^.

### 3.2 Optimization of experimental condition

**Fig. 4:**
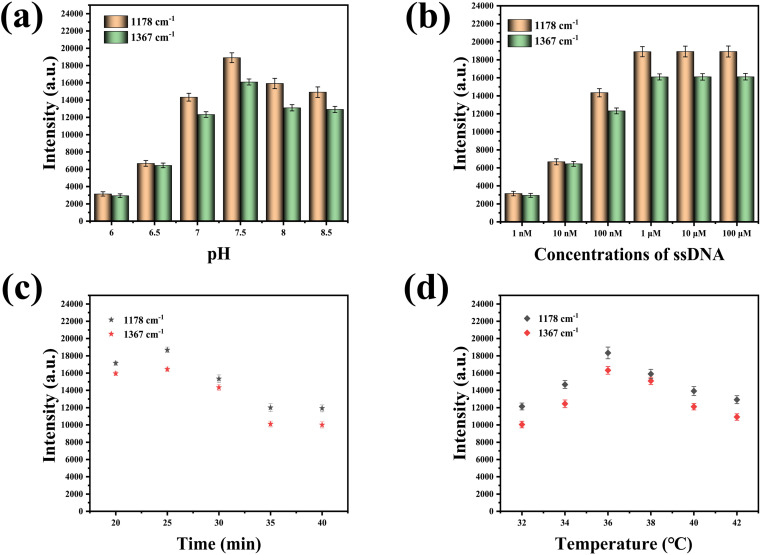
(a) Optimization of pH value, (b) optimization of ssDNA concentration, (c) optimization of detection time, (d) optimization of temperature.

To achieve optimal detection outcomes, experimental parameters, including pH, ssDNA concentration, detection duration, and temperature, had been meticulously optimized. As depicted in Fig. 4(a), the maximum SERS intensity had been recorded at a pH of 7.5. Fig. 4(b) illustrated that the characteristic peak intensities of 5-FAM at 1178 cm^−1^ and Cy5 at 1367 cm^−1^ had escalated with the increment of ssDNA concentration, plateauing when the concentrations of ssDNA-21 and ssDNA-25 reached 1 μM. This observation was ascribed to the saturation of ssDNA assembly on the AuNT array. A mixture of miR-21 and miR-25 had been incrementally introduced to the prepared SERS sensor to facilitate the formation of stable miR-21–ssDNA-21 and miR-25–ssDNA-25 duplexes. Subsequently, the DSN solution had been incrementally added, and SERS detection had been performed every 5 min to ascertain the most efficacious detection interval. Fig. 4(c) demonstrated that the intensities of the characteristic peaks at 1178 cm^−1^ and 1367 cm^−1^ had progressively diminished with the extension of the detection period, with the SERS intensity becoming relatively stable after 35 min. This trend was attributed to the complete enzymatic cleavage of miR-21–ssDNA-21 and miR-25–ssDNA-25 duplexes on the SERS sensor by DSN, which relocated the signal molecule 5-FAM away from the SERS sensor surface, culminating in a diminished SERS signal. Consequently, a detection duration of 35 min was identified as the most favorable for the assay. Ultimately, the experimental temperature was optimized to ascertain the most conducive conditions for the assay, as evidenced in Fig. 4(d), where the highest SERS intensity was recorded at 36 °C. As a result, 36 °C was determined to be the ideal temperature for detection.

### 3.3 Evaluation of the specificity and reproducibility of SERS sensors

**Fig. 5:**
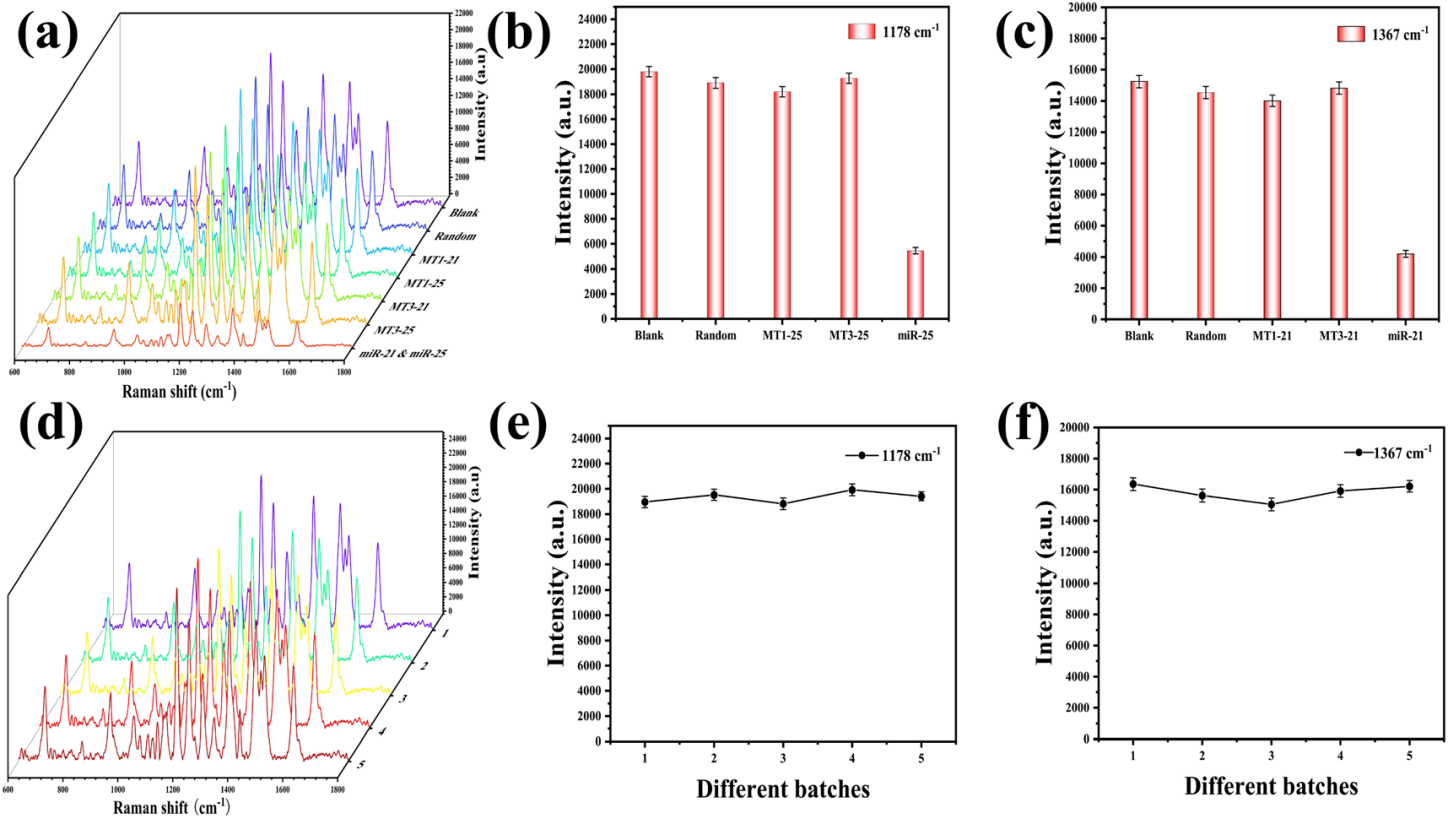
(a) SERS spectra obtained by reacting SERS sensors with different targets (blank, random, MT1-21, MT1-25, MT3-21, MT3-25, miR-21-miR-25), (b) SERS intensity at 1178 cm^−1^, (c) SERS intensity at 1367 cm^−1^, (d) SERS sensors prepared in different batches were used to detect the SERS spectrum of the same concentration sample, (e) SERS intensity at 1178 cm^−1^ for different batches of SERS sensors, (f) SERS intensity at 1367 cm^−1^ for different batches of SERS sensors.

The specificity and reproducibility of the SERS sensor were rigorously assessed under optimized experimental parameters. Within the scope of this investigation, specificity was determined by using MT1-21, MT1-25, MT3-21, MT3-25, and random sequences at 1 pM as a control group to determine the specificity of the SERS sensor. Despite the nucleotide sequence disparities among distinct miRNAs, the Raman reporter's characteristic peaks were substantially more pronounced than those of the miRNAs, attributable to the low concentration. This disparity resulted in the SERS spectra predominantly reflecting the Raman reporter molecule, with the miRNA-specific peaks being notably indistinct. As a consequence, the SERS spectra post-reaction with various targets exhibited a high degree of similarity. The resultant SERS spectra are depicted in Fig. 5(a), with the intensities of the characteristic peaks at 1178 cm^−1^ and 1367 cm^−1^ delineated in Fig. 5(b) and (c), respectively. In comparison with the control group, the characteristic peak intensities for miR-21 and miR-25 were markedly diminished at 1178 cm^−1^ and 1367 cm^−1^, suggesting a selective interaction of the SERS sensor with miR-21 and miR-25. Thus, the SERS sensor demonstrated a high degree of specificity in detection. To ascertain the reproducibility of the SERS sensor, a series of five SERS sensor batches were fabricated to detect an equimolar mixture of miR-21 (1 pM) and miR-25 (1 pM). The SERS spectra across different batches are presented in Fig. 5(d), with Fig. 5(e) and (f) illustrating the characteristic peak intensities at 1178 cm^−1^ and 1367 cm^−1^, respectively, which underscore the sensor's reproducibility. The relative standard deviation (RSD) values for the data presented in Fig. 5(e) were 2.32%, and for Fig. 5(f) were 3.28%.

### 3.4 Quantitative detection of miR-21 and miR-25

**Fig. 6:**
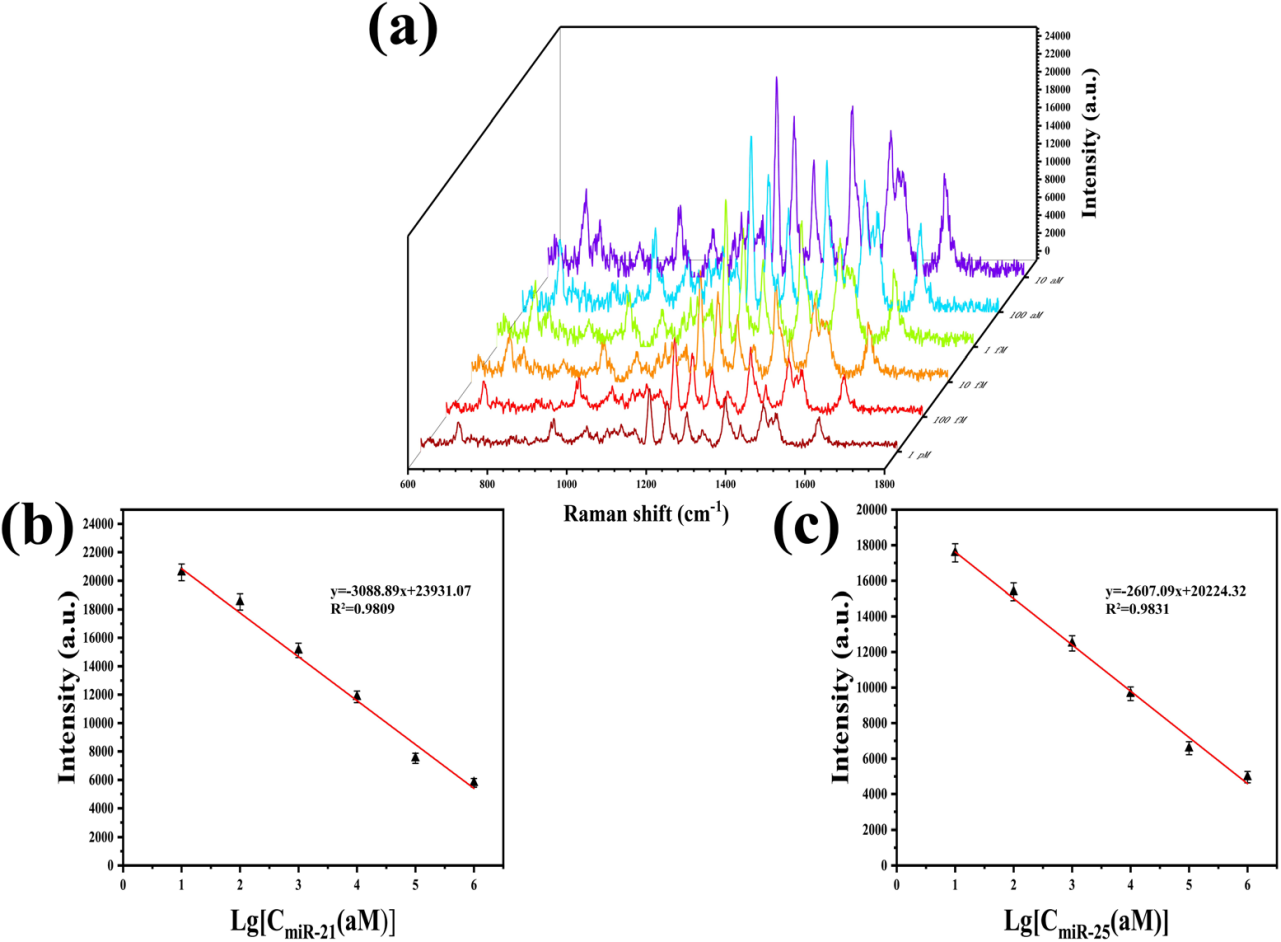
(a) SERS spectra obtained by reacting SERS sensors with different concentrations of miR-21 and miR-25 mixed solutions (10 aM, 100 aM, 1 fM, 10 fM, 100 fM, 1 pM), (b) linear equation of the logarithm of the concentration of miR-21 *versus* the SERS intensity at 1367 cm^−1^, (c) linear equation of the logarithm of the concentration of miR-25 *versus* the SERS intensity at 1178 cm^−1^.

The prepared SERS sensor was used to detect a mixture of miR-21 and miR-25 at various concentrations (10 aM, 100 aM, 1 fM, 10 fM, 100 fM, 1 pM). In order to achieve a range of distinct concentrations, we employed RNase-free water for serial dilutions. As depicted in Fig. 6(a), the SERS spectra were obtained by reacting the SERS sensor with mixed solutions of miR-21 and miR-25 at various concentrations. The characteristic peak intensities of Cy5 at 1367 cm^−1^ and 5-FAM at 1178 cm^−1^ gradually decreased with the increase in concentrations of miR-21 and miR-25. As illustrated in Fig. 6(b), the SERS intensity was linearly related to the logarithm of miR-21 concentration within the range of 10 aM to 1 pM. The linear regression equation was *y* = −3088.89*x* + 23 931.07, and the correlation coefficient (*R*^2^) was 0.9809. As shown in Fig. 6(c), the SERS intensity was linearly associated with the logarithm of miR-25 concentration over the concentration range of 10 aM to 1 pM. The equation for linear regression was *y* = −2607.09*x* + 20 224.32, and *R*^2^ was 0.9831. The limits of detection (LOD) for miR-21 and miR-25 in the SERS sensor were determined to be 4.29 aM and 8.12 aM, respectively. According to Table 2, the approach described in this paper exhibits a lower LOD in comparison to the alternative methods.

**Table 2:** Comparison of SERS with other miRNAs detection methods

Method	miRNA	Linear range	LOD	Ref.
Electrochemical	miR-21	100 aM–100 pM	77 aM	36
Fluorescence	miR-21	0.12 nM–1.2 nM	18 pM	37
SERS	miR-21	10 fM–10 nM	10 fM	38
SERS	miR-155	0.1 fM–100 pM	0.083 fM	39
SERS	miR-21	10 aM–1 pM	4.29 aM	This work
	miR-25		8.12 aM	

### 3.5 Serum sample testing

**Fig. 7:**
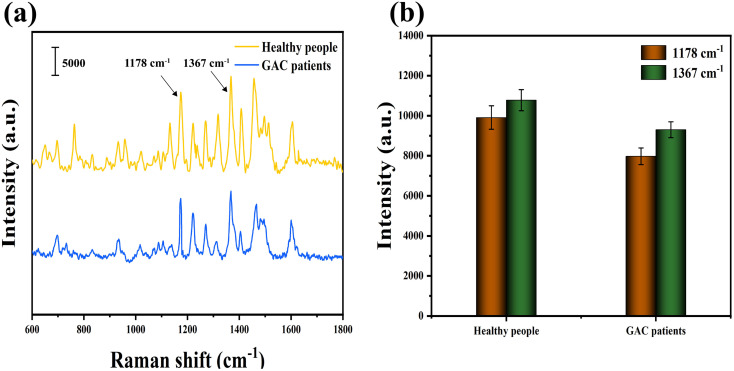
(a) Mean SERS spectra from serum from healthy people and GAC patients measured by SERS sensors, (b) SERS intensity at 1178 cm^−1^ and 1367 cm^−1^.

All enrolled subjects had been selected by gastroscopy and pathological examination. According to the HMDD database, the reference values for miR-21 in gastric cancer patients and healthy people are approximately 55 fM and 20 fM.^40^ The relative expression levels of miR-25 in the serum of GC patients or healthy controls are 0.47 ± 0.53 and 0.05 ± 0.36, respectively.^41^ Pathological photographs of all subjects were shown in Fig. S1 and S2.† To validate the feasibility of SERS technology for clinical application, the expression levels of miR-21 and miR-25 in serum samples from healthy people and GAC patients were measured. The mean SERS spectra of miR-21 and miR-25 in serum samples from 30 healthy individuals and 30 GAC patients are shown in Fig. 7(a), corresponding to the characteristic peak intensities at 1178 cm^−1^ and 1367 cm^−1^, as shown in Fig. 7(b). The concentrations of miR-21 and miR-25 were calculated by substituting the SERS intensity into a linear equation and comparing it to the real-time qRT-PCR results. As shown in Table 3, the relative errors of the two methods miR-21 in healthy people and gastric adenocarcinoma patients were 1.71% and −2.40%. The relative errors of miR-25 were 2.74% and −2.67%. SERS and qRT-PCR measured the concentrations of miR-21 and miR-25 in blood samples from 30 healthy individuals and GAC patients, as shown in Tables S1 and S2.† According to the formula: positive detection rate = [number of true positive samples/(number of false negative samples + number of true positive samples)] × 100% and negative detection rate = [number of true negatives/(number of false positives + number of true negative samples)] × 100%, we calculated the corresponding positive detection rate of 93.33% and negative detection rate of 96.67%. These results demonstrate that the method was highly reliable for the early diagnosis of GAC in clinical samples.

**Table 3:** SERS and qRT-PCR were used to detect the expression levels of miR-21 and miR-25 in serum samples

Sample	SERS (fM)		qRT-PCR (fM)		Relative error (%)	
	miR-21	miR-25	miR-21	miR-25	miR-21	miR-25
Health people	18.39	10.12	18.08	9.85	1.71	2.74
GAC patients	56.52	52.42	57.91	53.86	−2.40	−2.67

## 4. Conclusion

In this paper, based on AuNT and enzyme cleavage strategy, a SERS sensor for the detection of miR-21 and miR-25 in the serum of GAC patients was successfully prepared. The size of the synthesized AuNT topography was uniform, and the single-layer AuNT assembly exhibited a large number of evenly distributed “hot spots”, leading to notable enhancement of the SERS signal. The specificity and reproducibility of the SERS sensor were then evaluated, and the results were satisfactory. Within the time frame of 10 aM to 1 pM, there was a strong linear correlation between the logarithm of miR-21 concentration and the SERS intensity at 1367 cm^−1^, with the equation *y* = −3088.89*x* + 23 931.07 (*R*^2^ = 0.9809), and an impressive LOD of 4.29 aM was achieved. The logarithm of miR-25 concentration had a good linear relationship with the SERS intensity at 1178 cm^−1^, *y* = −2607.09*x* + 20 224.32 (*R*^2^ = 0.9831), and the LOD was as low as 8.12 aM. The results of this method showed no significant difference compared to qRT-PCR, but the expression levels of miR-25 and miR-21 were found to be higher in the serum of GAC patients than in healthy individuals.

## Data availability

We confirm that the data supporting the findings of this study are available within the main article and ESI.†

## Conflicts of interest

There are no conflicts to declare.

## Supplementary Material

RA-015-D4RA08761E-s001
