# Supplementary material for: Sensitive detection of miR-21 and miR-25 in gastric adenocarcinoma patient serum using a SERS sensor based on AuNT and enzyme cleavage strategy
Source: RSC Adv. 2025 Feb 10;15(6):4421–30. doi: 10.1039/d4ra08761e (PMC11808354; doi:10.1039/d4ra08761e)
Supplement: RA-015-D4RA08761E-s001 [file RA-015-D4RA08761E-s001.pdf]

Supplementary Information (SI) for RSC Advances.
This journal is © The Royal Society of Chemistry 2025

Supporting Information

Sensitive Detection of miR-21 and miR-25 in Gastric adenocarcinoma Patient Serum Using a SERS Sensor Based on AuNT and Enzyme Cleavage Strategy

Gaoyang Chen[2#], Ming Tan[4#], Long Jia[5], Yayun Qian[5], Hongjun Yin[1]*, Jinhua Zhu[3]*

[1]Department of Gastroenterology, Yangzhong People's Hospital, Zhenjiang 212200, P. R. China.

[2]Department of Oncology, The Affiliated Taizhou Second People's Hospital of Yangzhou University, Taizhou 225300, China

[3]Institute of Tumour Prevention and Control, Yangzhong People's Hospital, Zhenjiang, 212200, P. R. China.

[4]Department of General Surgery, Yangzhong People's Hospital, Zhenjiang 212200, P. R. China.

[5]Institute of Translational Medicine, Medical College, Yangzhou University, Yangzhou, 225001, P. R. China.

[#]Both authors contributed equally to this work.

*Correspondence: yzrykjk@163.com and 541292188@qq.com

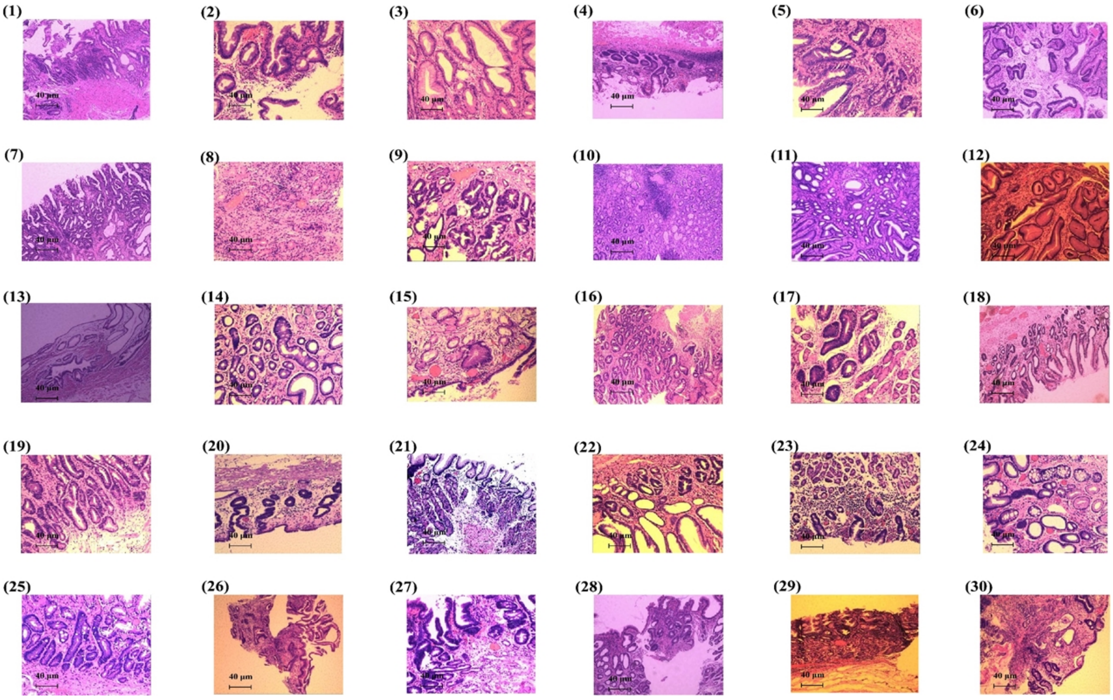

Fig.S1 Pathological pictures of 30 healthy people.

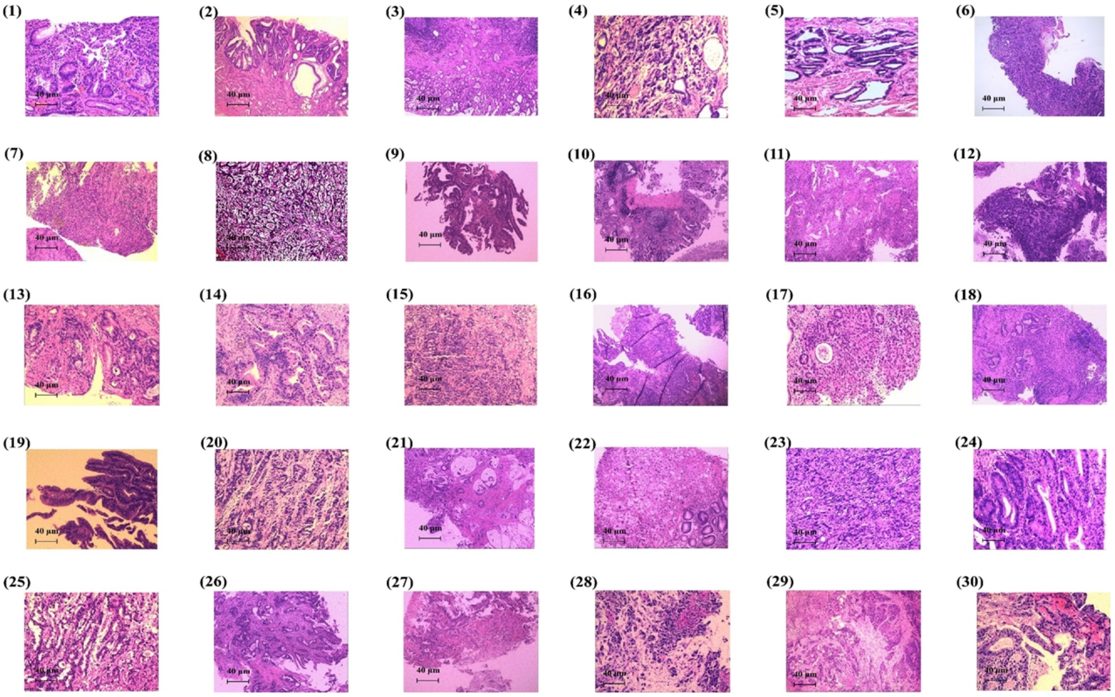

Fig.S2 Pathological pictures of 30 GAC patients.

Table S1 SERS and qRT-PCR were used to detect the expression levels of miR-21 and miR-25 in blood samples from 30 healthy people

Sample	SERS (fM)		qRT-PCR (fM)		Relative error (%)	
	miR-21	miR-25	miR-21	miR-25	miR-21	miR-25
1	17.54	9.52	18.05	9.15	-2.83	4.04
2	18.28	10.48	17.85	10.89	2.41	-3.76
3	18.37	15.21	18.91	15.85	-2.86	-4.04
4	18.42	6.28	17.86	6.42	3.14	-2.18
5	18.35	17.21	18.93	17.64	-3.06	-2.44
6	18.24	7.17	18.94	6.94	-3.7	3.31
7	18.19	6.89	18.68	6.62	-2.62	4.08
8	18.42	5.21	18.98	5.02	-2.95	3.78
9	18.45	14.28	18.86	13.95	-2.17	2.37
10	18.48	13.95	19.05	14.25	-2.99	-2.11
11	19.32	8.54	18.63	8.15	3.7	4.79
12	19.59	13.08	18.98	12.75	3.21	2.59
13	17.13	5.18	16.45	5.02	4.13	3.19
14	17.25	3.55	16.96	3.68	1.71	-3.53
15	35.35	29.28	26.22	20.11	34.82	45.6
16	10.26	8.69	10.05	9.02	2.09	-3.66
17	15.69	6.67	16.18	6.85	-3.03	-2.63
18	14.39	15.01	14.06	14.85	2.35	1.08
19	19.83	8.95	20.25	8.61	-2.07	3.95
20	20.53	5.28	21.05	5.08	-2.47	3.94
21	18.89	6.37	18.21	6.12	3.73	4.08
22	18.99	7.06	18.54	7.15	2.43	-1.26
23	19.34	7.38	18.91	7.13	2.27	3.51
24	20.12	6.91	19.76	6.61	1.82	4.54
25	18.54	5.08	17.92	5.14	3.46	-1.17
26	18.39	10.25	17.63	10.66	4.31	-3.85
27	16.85	14.15	17.02	14.52	-1	-2.55
28	14.85	8.45	14.13	8.84	5.1	-4.41
29	17.54	8.39	18.25	8.81	-3.89	-4.77
30	16.25	19.24	17.07	19.55	-4.8	-1.59

Table S2 SERS and qRT-PCR were used to detect the expression levels of miR-21 and miR-25 in blood samples from 30 GAC patients

Sample	SERS (fM)		qRT-PCR (fM)		Relative error (%)	
	miR-21	miR-25	miR-21	miR-25	miR-21	miR-25
1	58.92	48.52	58.02	50.12	1.55	-3.19
2	16.85	19.25	30.45	32.85	-44.66	-41.4
3	59.67	65.19	60.55	63.15	-1.45	3.23
4	60.79	31.35	58.48	30.48	3.95	2.85
5	68.59	59.37	72.81	62.41	-5.8	-4.87
6	52.14	64.93	54.06	61.85	-3.55	4.98
7	62.78	68.19	60.78	66.12	3.29	3.13
8	59.48	60.86	57.91	58.75	2.71	3.59
9	68.27	79.14	70.76	77.18	-3.52	2.54
10	33.85	60.54	35.16	57.92	-3.73	4.52
11	66.48	40.19	69.55	41.85	-4.41	-3.97
12	62.15	53.85	60.47	55.72	2.78	-3.36
13	58.34	46.85	60.72	48.88	-3.92	-4.15
14	57.15	70.18	54.76	72.51	4.36	-3.21
15	56.37	60.83	58.62	63.15	-3.84	-3.67
16	59.15	62.83	61.86	60.11	-4.38	4.53
17	68.21	34.19	65.85	35.94	3.58	-4.87
18	53.11	42.85	50.94	44.93	4.26	-4.63
19	67.19	58.63	70.08	57.18	-4.12	2.54
20	59.84	40.18	57.15	41.99	4.71	-4.31
21	54.37	55.63	52.02	53.08	4.52	4.8
22	58.21	51.08	56.74	52.57	2.59	-2.83
23	20.18	18.26	39.45	31.28	-48.85	-41.62
24	58.91	65.18	61.85	68.15	-4.75	-4.36
25	71.52	42.81	73.25	44.75	-2.36	-4.34
26	65.84	55.65	67.72	58.14	-2.78	-4.28
27	51.08	48.24	53.45	50.28	-4.43	-4.06
28	55.31	47.28	57.24	49.28	-3.37	-4.06
29	54.05	56.35	52.46	59.18	3.03	-4.78
30	56.82	64.18	54.18	66.19	4.87	-3.04
